# Evaluation of different analysis pipelines for the detection of HIV-1 minority resistant variants

**DOI:** 10.1371/journal.pone.0198334

**Published:** 2018-06-01

**Authors:** Marine Perrier, Nathalie Désiré, Alexandre Storto, Eve Todesco, Christophe Rodriguez, Mélanie Bertine, Quentin Le Hingrat, Benoit Visseaux, Vincent Calvez, Diane Descamps, Anne-Geneviève Marcelin, Charlotte Charpentier

**Affiliations:** 1 IAME, UMR 1137, INSERM, Université Paris Diderot, Sorbonne Paris Cité, AP-HP, Laboratoire de Virologie, Hôpital Bichat, AP-HP, Paris, France; 2 Sorbonne University, UPMC Univ Paris 06, INSERM, Institut Pierre Louis d'épidémiologie et de Santé Publique (IPLESP UMRS 1136), Paris, France; 3 Hôpital Pitié Salpêtrière, Laboratoire de Virologie, Paris, France; 4 Département de Microbiologie, Next-Generation Sequencing Platform pACT, IMRB Créteil, Créteil, France; 5 Institut Mondor de Recherche Biomédicale U955, Créteil, France; University of Pittsburgh, UNITED STATES

## Abstract

**Objective:**

Reliable detection of HIV minority resistant variants (MRVs) requires bioinformatics analysis with specific algorithms to obtain good quality alignments. The aim of this study was to analyze ultra-deep sequencing (UDS) data using different analysis pipelines.

**Methods:**

HIV-1 protease, reverse transcriptase (RT) and integrase sequences from antiretroviral-naïve patients were obtained using GS-Junior^®^ (Roche) and MiSeq^®^ (Illumina) platforms. MRVs were defined as variants harbouring resistance-mutation present at a frequency of 1%–20%. Reads were analyzed using different alignment algorithms: Amplicon Variant Analyzer^®^, Geneious^®^ compared to SmartGene^®^ NGS HIV-1 module.

**Results:**

101 protease and 51 RT MRVs identified in 139 protease and 124 RT sequences generated with a GS-Junior^®^ platform were analyzed using AVA^®^ and SmartGene^®^ software. The correlation coefficients for the MRVs were R^2^ = 0.974 for protease and R^2^ = 0.972 for RT. Discordances (n = 13 in protease and n = 15 in RT) mainly concerned low-level MRVs (i.e., with frequencies of 1%–2%, n = 18/28) and they were located in homopolymeric regions (n = 10/15). Geneious^®^ and SmartGene^®^ software were used to analyze 143 protease, 45 RT and 26 integrase MRVs identified in 172 protease, 69 RT, and 72 integrase sequences generated with a MiSeq^®^ platform. The correlation coefficients for the MRVs were R^2^ = 0.987 for protease, R^2^ = 0.995 for RT and R^2^ = 0.993 for integrase. Discordances (n = 9 in protease, n = 3 in RT, and n = 3 in integrase) mainly concerned low-level MRVs (n = 13/15).

**Conclusion:**

We found an excellent correlation between the various UDS analysis pipelines that we tested. However, our results indicate that specific attention should be paid to low-level MRVs, for which the use of two different analysis pipelines and visual inspection of sequences alignments might be beneficial. Thus, our results argue for use of a 2% threshold for MRV detection, rather than the 1% threshold, to minimize misalignments and time-consuming sight reading steps essential to ensure accurate results for MRV frequencies below 2%.

## Introduction

Human Immunodeficiency Virus (HIV) sequencing is used to detect resistance-associated mutations (RAMs) at the time of virological failure to assess acquired drug resistance as well as at the time of diagnosis to assess transmitted drug resistance [1]. Routine HIV sequencing is currently performed using Sanger technology, which can detect majority viral variants; i.e., those representing more than 20% of the total viral population [2,3]. Recently developed ultra-deep sequencing (UDS) technologies produce a massive data volume and they are able to detect minority viral variants that harbor RAMs down to a frequency of 1% [2,4,5]. The detection of transmitted drug resistance is increased by a factor of 2 to 3 by the use of UDS technologies compared to Sanger technology [6–8]. Similarly, the use of UDS also increases the number of RAMs detected at the time of virological failure [9,10].

Most of the studies to date using UDS technologies define a frequency of 1% as the threshold for detection of minority resistant variants (MRVs) [6,9,11,12]. However, a good alignment quality generated by bioinformatics analysis software for UDS reads is mandatory for reliable detection and/or quantification of MRVs, especially when they occur at low frequencies. Several software alignment algorithms are available to analyze UDS data. These are, however, not necessarily specifically designed for HIV MRV analysis, and the analysis of HIV MRVs can present problems for alignment algorithms due to the existence of viral quasispecies and the presence of several homopolymeric regions along the viral genome. Currently, there are two licensed commercial software programs specifically dedicated to the analysis of HIV MRV: DeepChek^^®^^-HIV [13] and SmartGene^^®^^ [14], while other non-licensed generalist analysis pipelines are also available [14].

This study analyzed the results of UDS data generated with GS Junior^®^ or MiSeq^®^ platforms from HIV-1 clinical samples using three different bioinformatics analysis pipelines: Amplicon Variant Analyzer (AVA^®^), SmartGene^®^ NGS HIV-1 CE-labeled (SmartGene, Zug, Switzerland) and Geneious^®^.

The aim of this study was to assess the correlation in the detection and/or quantification of HIV MRVs using different bioinformatics analysis pipelines, in order to validate the analytical steps of bioinformatics data management for the purpose of future UDS routine use.

## Material and methods

### HIV *pol* sequences

We assessed HIV type 1 (HIV-1) protease, reverse transcriptase (RT) and integrase sequences in samples collected from antiretroviral-naïve patients.

Some of the sequences were obtained from PCR amplicons using a GS Junior^®^ platform (Roche 454 Life Sciences, Branford, CT, USA), according to the procedures of the French Agency for Research on AIDS and Viral Hepatitis (ANRS) (www.hivfrenchresistance.org). Another portion of the sequences was obtained from PCR amplicons using a MiSeq^®^ platform (Illumina, San Diego, CA, USA), according to the manufacturer’s recommendations.

Briefly for GS Junior^®^ platform, after RNA extraction (NucliSENS^®^ Easy MAG, bioMérieux Clinical Diagnostic) and reverse-transcription (Titan One Tube RT-PCR Kit^®^, Roche Applied Science) [10,15], a nested-PCR was made with a high-fidelity Taq polymerase (Q5^®^ High-Fidelity DNA Polymerase, New England Biolabs). PCR products were purified by AMPure^®^ Beads (Agencourt, Biosciences), quantified using Qubit^®^ 2.0 Fluorometer (Life Technologies) and pooled equimolarly. Pyrosequencing on the GS Junior^®^ (Roche 454^®^ Life Sciences) was performed according to the manufacturer’s recommendations. Ultra-Deep Sequencing of RT region was performed in 2 fragments: RT1 (RT amino acid 17–140) and RT2 (RT amino acid 133–247).

With Illumina^®^ technology, HIV-1 RNA was extracted using EZI Virus Mini Kit^®^ v2.0 (Qiagen, Hilden, Germany) and amplified by PCR using SuperScript^®^ III One-Step RT-PCR System with Platinum^®^ Taq High Fidelity (Thermofisher, Waltham, MA, USA). Protease, RT and integrase regions were amplified as a single amplicon only, encompassing the whole *pol* gene using the following outer and inner primers [16], respectively: Pan-HIV-1_2F (5’-GGGAAGTGAYATAGCWGGAAC) PCRPOL-R (5’-TATGGAGACYCCMTGACC) and NPCRPOL-F (5’-GACAGCATGYCAGGGAG)/NPCRPOL-R (5’-TGGGATRTGTACTTCTGARC). A 3385 bp fragment was then amplified by a second round of PCR using PrimeSTAR^®^ GXL DNA Polymerase Kit (Takara Bio Inc., Nojihigashi, Japan) with the primers NPCRPOL-F (5’-GACAGCATGYCAGGGAG) and NPCRPOL-R (5’-TGGGATRTGTACTTCTGARC). PCR products were purified by AMPure^®^ Beads (Agencourt, Biosciences), quantified using Qubit^®^ 2.0 Fluorometer (Life Technologies) and pooled equimolarly. UDS was performed using Illumina Miseq^®^ technology (Illumina, San Diego,CA, USA.

The GS Junior^®^ and MiSeq^®^ platforms generated Standard Flowgram Files (sff) and fastq files, respectively.

Protease, RT and integrase resistance mutations were identified according to the resistance algorithm developed by the ANRS (www.hivfrenchresistance.org, version 26). We only considered variants that had a frequency greater than 1%. In this study, we defined an MRV as a variant carrying a RAM present in 1%-20% of the viral population.

### Alignment software

The same reads were analyzed using three different analysis pipelines, all of which are dedicated to bioinformatics analysis of UDS data: (i) Amplicon Variant Analyzer (AVA^®^), a software directly integrated in the GS Junior^®^ platform; (ii) SmartGene^®^ NGS HIV-1 module (SmartGene, Zug, Switzerland), a software specifically developed for the detection of HIV MRVs; and (iii) Geneious^®^ research software (version 9.0.5), which is a more generalist alignment software.

#### Amplicon Variant Analyzer (AVA^®^)

The flowgram format (sff) data files were analyzed using AVA^®^ 2.7 software with the HXB2 HIV-1 reference sequence (GenBank accession n^o^ K03455). The reads were trimmed using a quality filter, demultiplexed based on the multiplex identifiers (MIDs) for each sample, and mapped to the HXB2 reference sequence. Variant calls were made using the default AVA^®^ settings. Variants were considered valid when present in both forward and reverse directions in a balanced manner as previously described [10]. All variants represented by at least 50 sequencing reads and at a frequency >1% from the reference sequence were selected. All alignments were carefully reviewed for alignment errors and analysis.

#### SmartGene^®^ NGS HIV-1 module

The SmartGene^®^ NGS HIV-1 module is based on the proprietary IDNS (Integrated Database Network System). This analysis platform offers a web-interface on which sff or fastq files can be uploaded. The analysis pipeline can be chosen by the user. Also appropriate cut-off for ambiguous bases/background (0.5%), minimum coverage of variant (50 reads) and interpretation cut-off have been selected before the analysis. All of the reads were demultiplexed, grouped by MIDs and trimmed by applying a high quality score filtering. Alignments of reads were performed against the HXB2 reference sequence and a particular homopolymers-related correction was applied on these specific regions. Viral variant frequencies were determined at each nucleotide position relative to the HXB2 reference sequence. A minimum of depth coverage of 50 reads were required to find variant nucleotide sequence. HIV drug resistance mutations were interpreted with the embedded ANRS resistance algorithm at the chosen cut-off (0.5–20%). The complete mutation list with their respective frequencies as percentage values was compiled, and lastly a drug resistance profile was generated for each sample.

#### Geneious^®^ version 9.0.5

Reads from Illumina^®^ sequencing were analyzed using Geneious^®^ version 9.0.5 software (Biomatters Ltd, Auckland, NZ) (http://www.geneious.com) [17]. A HXB2 reference sequence (GenBank Accession n^o^ K03455) was uploaded into Geneious^®^ and all drug resistance mutations were manually annotated onto the reference sequence for reference-based assembly. Sequences were demultiplexed automatically on the MiSeq^®^ platform as part of the data processing steps and two paired .fastq files were generated for each sample representing the two paired-end reads. After importing the .fastq files into Geneious^®^, the two sequence lists from each sample were paired. Sequences were then mapped to the annotated reference sequence with Geneious^®^ Read Mapper with medium sensitivity and 5 fine-tuning iteration parameters. All variants represented by at least 50 sequencing reads and at a frequency >1% from the reference sequence were selected. All detected variants were present in both forward and reverse directions in a balanced manner as previously described [10]. The frequency of each variant and the number of sequences representing each nucleotide position containing a variant different from the reference sequence were also calculated by the variant finder Variants and their frequencies were exported into an Excel^®^ document and filtered for those that occurred at amino acids associated with drug resistance.

Read alignments were typically visually verified in two situations: (i) when an MRV was detected by only one of the software programs; (ii) when an MRV was detected by both software programs, albeit with a difference in the MRV quantification of more than 2% if the MRV frequency was 1%-10%, and more than 5% if the MRV frequency was 10%-20%.

### Ethics statement

The sequences used in this study were obtained as part of the patients’ routine follow-up at the Bichat-Claude Bernard and the Pitié-Salpêtrière hospitals, and they are not accessible to other researchers for the purpose of replication. As the sequence data were anonymized as soon as they were obtained, only anonymized sequences were assessed in this study. The laboratories involved in this study belong to the ANRS and they participate in the ANRS quality control assessment of HIV-1 drug resistance sequencing. This study was approved by the scientific committee of the ANRS *Action Coordonnée* n°11.

### Statistical analysis

Linear regression slopes were plotted to compare MRV frequency between the analysis software and correlation coefficients were calculated with Pearson's test. Bland-Altman plots were used to assess the level of agreement between the analysis software by plotting the percentage of difference in MRV frequencies against the average of the two measurements [18]. Bland-Altman recommended that 95% of the data points should lie within ±1.96 standard deviation (SD) of the mean difference. All this calculation were plotted using R 3.3.2 [19]

## Results

### Analysis using AVA^®^ and SmartGene^®^ software

In this analysis of sff data files obtained with a GS Junior^®^ platform, 139 protease and 124 RT HIV-1 sequences were assessed, in which 101 and 51 MRVs were detected, respectively. Overall, MRVs were present at a median frequency of 2.5% (IQR = 1.3–5.5). A median of 5,198 and 6,691 reads were mapped to the reference sequence for protease and RT regions, respectively.

### Protease analysis

At a detection threshold of 1%, differences in MRV detection and/or quantification between the two software programs were observed for 13 MRVs (9.4%) in the protease region (Table 1). Most of these differences (n = 9/13) concerned low-level MRV (i.e., those with frequencies of 1%-2%), which were detected by only one of the software programs. In two of these nine cases, the MRVs were detected with a frequency of 0.8%-1.0% by AVA^®^, thus revealing no significant difference with the 1.1% frequency of both variants as detected by SmartGene^®^. In two other cases, the MRVs were detected with a frequency above 2% by one of the software programs and not by the other (i.e., a 3.9% frequency of A71T by SmartGene^®^ and a 2.3% frequency of G73A by AVA^®^). Regarding the A71T MRV, visual inspection of the AVA^®^ read alignments revealed misalignments, and manual correction of the read alignments led to a frequency of 3.8% for this MRV, which is very close to the frequency of 3.9% detected by SmartGene^®^. For the G73A MRV, the difference can be explained by the low level of coverage at this position, which was below 1,000 reads with both software programs. Finally, for the two remaining differences, both MRVs were detected by the two software programs, although with differences in terms of quantification: L10I at a frequency of 4.6% by AVA^®^ versus a frequency of 1.2% by SmartGene^®^, and M46I at a frequency of 7.0% by AVA^®^ versus a frequency of 3.6% by SmartGene^®^. In both cases, the coverage was low (i.e., < 2,000 reads) as a result of stringent quality filters in the SmartGene^®^ analysis, since the M46I mutation is located right after a stretch of poly-A sequence.

**Table 1:** AVA^®^ and SmartGene^®^ analyses.

Mutation	AVA^®^	SmartGene^®^
**PROTEASE**		
I15V	<1% (Not detected)	1.6%
I15V	<1% (Not detected)	1.3%
I15V	<1% (Not detected)	1.1%
K20I	< 1% (0.9%)	1.1%
I62V	<1% (Not detected)	1.2%
H69Y	<1% (Not detected)	1.5%
L10I	4.5%	1.2%
L89R	1.7%	<1% (Not detected)
A71T	<1% (Not detected)*	3.9%
A71V	<1% (Not detected)	1.1%
M46I	7.0%	3.6%
G73A	2.3%	<1% (Not detected)
V82A	< 1% (0.9%)	1.1%
**REVERSE TRANSCRIPTASE**		
E44D	<1% (Not detected)	1.3%
K65E	<1% (Not detected)	1.2%
K65E	<1% (0.9%)	2.5%
K65E	25.7%*	1.4%
T69S	<1% (0.9%)	3.6%
L100I	<1% (Not detected)	1.6%
K101E	<1% (Not detected)	3.0%
K101R	9.8%*	29.8%
V179M	<1% (Not detected)	1.2%
Y181C	1.1%	<1%(Not detected)
K101I	<1% (0.6%)	1.2%
K101R	<1% (Not detected)	1.2%
K103R	<1% (Not detected)	1.3%
V179D	<1% (Not detected)*	7.0%
K101E	<1% (0.8%)	1.2%

*After manual correction, the detection/quantification of the minority resistant variant with the AVA^®^ software was found to occur at a similar frequency as with the SmartGene^®^ analysis.

At a threshold of 2% for MRV detection (Table 2), only four discrepancies in the protease region remained between the two analysis pipelines, and one (MRV A71T) could be corrected as a result of the manual review, as described above. Thus, only three differences (2.9%) remained among the 101 MRVs that were compared using the two software programs.

**Table 2:** The number of discordances between the analysis pipelines according to the variant detection threshold used for the ultra-deep sequencing analyses.

Analysis pipelines	Number of discordances, n (%)	
	1% threshold	2% threshold
AVA^®^ *versus* SmartGene^®^	28 (18.4%)	8 (5.3%)
Geneious^®^ *versus* SmartGene^®^	15 (7.0%)	2 (0.9%)

**Fig 1:**
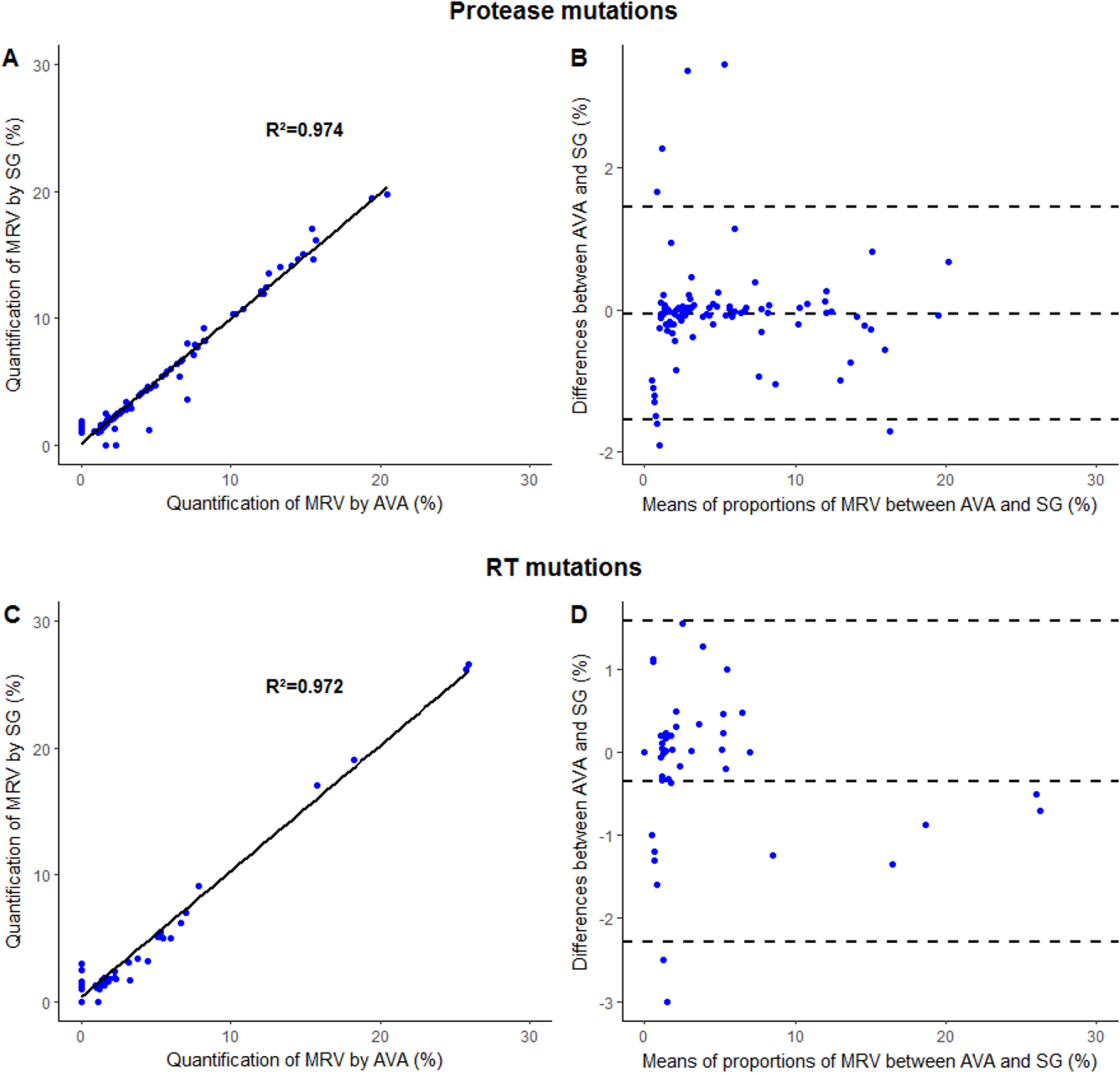
**Comparison of the quantification of minority resistant variants (MRV) using SmartGene^®^ (SG) and AVA^®^ pipeline analysis for protease mutations (A,B) and Reverse Transcriptase (RT) (C, D) mutations after excluding two major discrepancies**. Minority resistant variants frequencies were depicted according to linear regression (A, C) and Bland-Altman (B, D) analyses. For Bland-Altman analysis, y-axis indicates the percentage of difference in MRV frequency measurements between AVA^®^ and SG^®^ analysis and x-axis indicates the mean of the two measurements. All MRV detected by both software were included in this analysis. The 95% confidence intervals limits of agreement were calculated. Mean, upper and lower limits of agreement were depicted by the dotted lines.

The correlation coefficient for the proportion of MRVs obtained by AVA^®^ analysis and those obtained by SmartGene^®^ analysis was R^2^ = 0.974 for the protease region (Fig 1). Bland-Altman plot analysis showed a very good agreement with only 7 data points (6.9%) outside the 95% confidence interval.

### RT analysis

In regard to RT mutations, at a detection threshold of 1%, differences in MRV detection and/or quantification between the two software programs were observed for 15 MRVs (29.4%), including five nucleoside RT inhibitor (NRTI) RAMs and 10 non-NRTI (NNRTI) RAMs (Table 1). Most of the discordant MRVs (n = 10/15, 75%) were located in homopolymeric regions. Most of the differences (n = 9/15) concerned low-level MRVs, with frequencies below 2%. These were detected by one of the software programs but not by the other. Among these cases, two MRVs were detected at a frequency below 1% (between 0.60% and 0.75%) by AVA^®^, thus revealing no significant difference with the 1.2% frequency of these variants with SmartGene^®^.

Major discrepancies between the software analyses were observed in two cases. The first one was observed at position 101 in one sample, for which the variant K101R was detected at a frequency of 9.8% by AVA^®^ compared to a frequency of 29.8% by SmartGene^®^. Visual inspection of the read alignments revealed reads with a single-nucleotide deletion in this codon. These reads with poor quality scores had been removed by the SmartGene^®^ software alignment algorithm, but not by the AVA^®^ one. Thus, differences in the data processing could explain this observed discrepancy. The second major discrepancy observed between the two software programs was at position 65, and it was again for a single sample: the variant K65E was detected at a frequency of 25.7% by AVA^®^ compared to a frequency of 1.4% by SmartGene^®^. Visual inspection of the read alignments by AVA^®^ revealed that the K65E reads were mainly present in reverse reads and that they had low Phred quality scores, which most likely led to an overestimation of the frequency of the K65E variant.

In four other cases, MRVs were detected at frequencies greater than 2% by one of the software programs while not being detected by the other: (i) one case was corrected after manual review (V179D), (ii) one case exhibited a low coverage below 1,000 reads (K101E), (iii) one displayed a correct alignment by the two software programs (T69S), and (iv) one was located in a homopolymeric region (K65E) that generated misalignment with AVA^®^.

At a threshold of 2% for MRV detection, only six discrepancies were observed between the two software programs. As described above, one (MRV V179D) was corrected after the manual review. Thus, at a detection threshold of 2%, only five differences (9.8%) remained between the software programs (Table 2).

The correlation coefficient for the proportion of MRVs obtained by AVA^®^ analysis and those obtained by SmartGene^®^ analysis was R^2^ = 0.602 for the RT region. However, since the two major discordant MRVs, at positions 65 and 101, were easily detected by the use of two different software programs, and since they resulted from AVA^®^ misalignments, we decided to exclude them for the final analysis, thereby leading to a correlation coefficient of R^2^ = 0.972 (Fig 1). Bland-Altman plot analysis showed a very good agreement with only 2 data points (3.9%) outside the 95% confidence interval.

By combining the protease and the RT reads, the AVA^®^ and the SmartGene^®^ analyses covered 152 MRVs, which yielded a good correlation coefficient of R^2^ = 0.95.

### Analysis using Geneious^®^ and SmartGene^®^ software

In this analysis of fastq data files obtained with a MiSeq^®^ platform, 172 protease, 69 RT, and 72 integrase HIV-1 sequences were assessed, in which 143, 45, and 26 MRVs were detected, respectively. Overall, MRVs occurred at a median frequency of 2.9% (IQR = 1.4–5.6). A median of 75,635, 14,588, and 87,210 reads were mapped to the protease, RT, and integrase region reference sequences, respectively.

#### Protease analysis

In regard to protease mutations, at a detection threshold of 1%, differences in MRV detection and/or quantification between the two software programs were observed for 9 MRVs (6.3%) (Table 3). Most of these (n = 7/9) resulted from the detection of low-level MRVs (i.e., with frequencies below 2%) by one of the software programs, whereas these MRVs were not detected by the second software program. Among these seven cases, six MRVs were found at a frequency below 1% (between 0.3% and 0.9%) with the second analysis pipeline, thus revealing no significant difference with the low-frequency MRVs detected by the other software. One case of discrepancy indicated a difference in MRV quantification, with the M46I MRV having a frequency of 1.4% by SmartGene^®^ versus a frequency of 5.7% by Geneious^®^ analysis. This difference resulted from a reduced number of reads in the SmartGene^®^ analysis compared to the Geneious^®^ analysis, which was due to a more stringent selection of reads with the SmartGene^®^ alignment. At the threshold of 2% for MRV detection, only two differences (1.4%) remained (Table 2).

**Table 3:** Geneious^®^ and SmartGene^®^ analyses.

Mutation	Geneious^®^	SmartGene^®^
**PROTEASE**		
L10I	<1% (0.9%)	1.2%
K20R	<1% (0.8%)	1.2%
M46I	5.7%	1.4%
M46I	2.2%	<1% (Not detected)
I62V	<1% (0.9%)	1.2%
L63P	1.1%	<1% (0.3%)
A71V	<1% (0.8%)	1.1%
V77I	1.2%	<1% (0.9%)
V82F	1.1%	<1% (Not detected)
**REVERSE TRANSCRIPTASE**		
K101R	1.1%	<1% (0.9%)
V106I	<1% (0.6%)	1.1%
P225H	1.4%	<1% (0.7%)
**INTEGRASE**		
L74M	1.2%	<1% (0.7%)
L74M	1.1%	<1% (0.6%)
L74M	1.1%	<1% (0.6%)

#### RT analysis

In regard to RT mutations, at a detection threshold of 1%, only three differences (6.7%) were observed, all concerning NNRTI RAMs (Table 3). All three of these cases concerned low-level MRVs, detected at frequencies of 1%-2% by one of the software programs, whereas they had a frequency below 1% (between 0.6% and 0.9%) with the second software program, thus not amounting to a significant difference with the other software program. At a threshold of 2% for MRV detection, no discrepancy was observed between the two software programs among the 69 RT MRVs that were assessed (Table 2).

#### Integrase analysis

In regard to the integrase region, at a threshold of 1% for MRV detection, only three MRVs (11.5%) were discordant, all of which were located at position 74 (Table 3). These three L74I MRVs had a frequency below 1% by SmartGene^®^, whereas they had a frequency of 1.1%-1.2% by Geneious^®^ analysis. However, when applying a detection threshold of 0.5% with the SmartGene^®^ software, these three MRVs had frequencies of 0.6%-0.7%, which are in a similar range to those detected with the Geneious^®^ software. At a threshold of 2% for MRV detection, no difference was observed between the two analysis pipelines among the 72 integrase MRVs that were assessed (Table 2).

**Fig 2:**
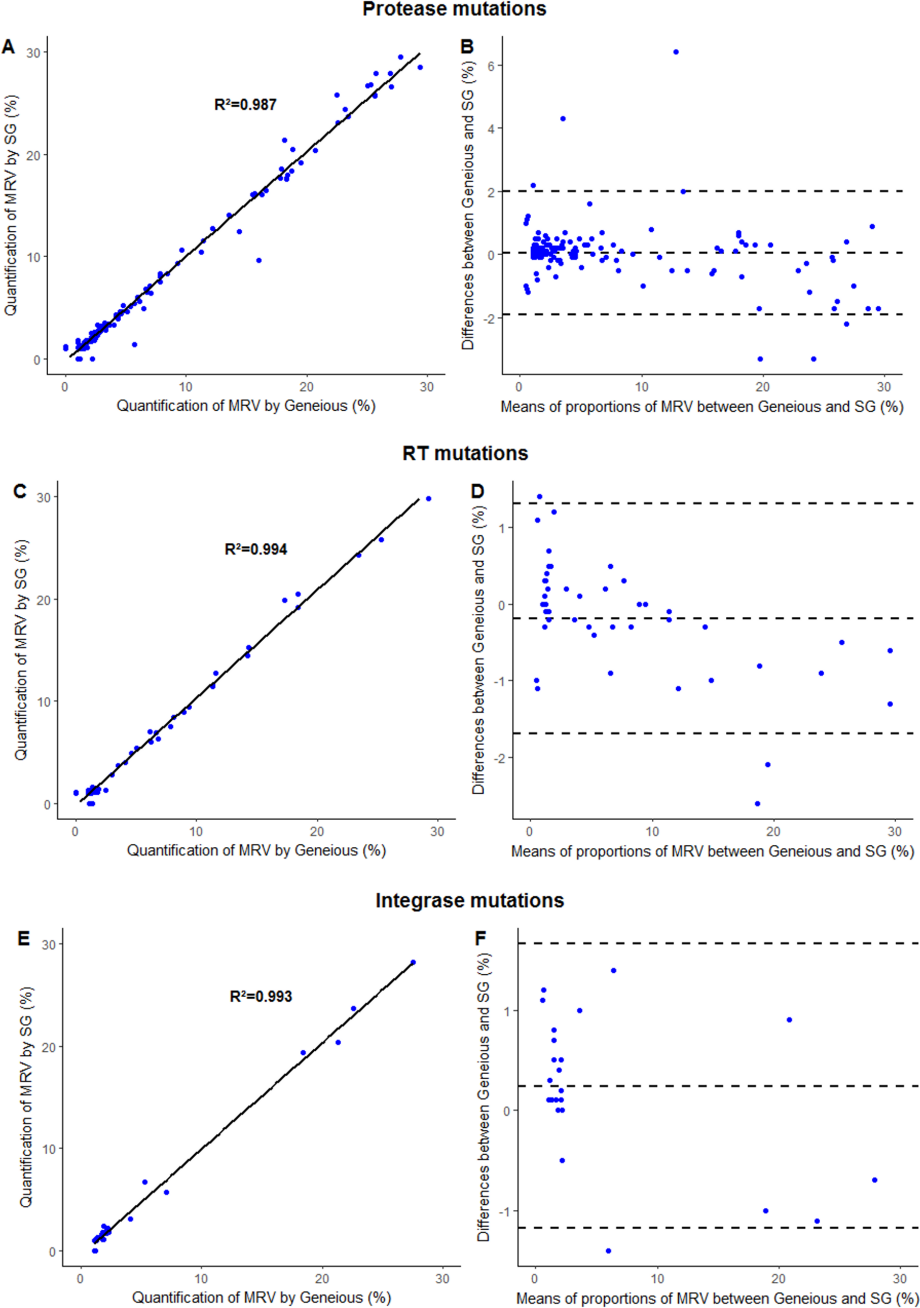
**Comparison of the quantification of minority resistant variants (MRV) using SmartGene^®^ (SG) and Geneious^®^ pipeline analysis for protease mutations (A, B), Reverse Transcriptase (RT) (C, D) and integrase mutations (E, F).** Minority resistant variants frequencies were depicted according to linear regression (A, C, E) and Bland-Altman (B, D, F) analyses. For Bland-Altman analysis, y-axis indicates the percentage of difference in MRV frequency measurements between Geneouis^®^ and SG^®^ analysis and x-axis indicates the mean of the two measurements. All MRV detected by both software were included in this analysis. The 95% confidence interval limits of agreement were calculated. Mean, upper and lower limits of agreement were depicted by the dotted lines.

The Geneious^®^ and SmartGene^®^ analyses covered 214 MRV, revealing an excellent correlation coefficient of R^2^ = 0.987 for protease, R^2^ = 0.995 for RT, and R^2^ = 0.993 for integrase (Fig 2). Regarding Bland-Altman plot analysis, a very good agreement was observed between Geneious^®^ and SmartGene^®^ analysis with only 6 (4.2%), 3 (6.7%) and 1 (3.8%) data points outside the 95% confidence interval for protease, RT and integrase regions, respectively.

## Discussion

In this study, we found that there was a good correlation between SmartGene^®^ and AVA^®^ as well as between SmartGene^®^ and Geneious^®^ pipeline analyses for the detection and quantification of MRVs using sequence data obtained with GS Junior^®^ and Illumina^®^ platforms, respectively.

Firstly, we compared AVA^®^ and SmartGene^®^ pipeline analyses for 152 HIV-1 MRVs. AVA^®^ is not an aligner tool meant for alignment of minority variants. By contrast, the SmartGene^®^ analysis pipeline was specifically designed with HIV quasispecies in mind, including alignment algorithms that take into account issues relating to homopolymeric regions and of quasispecies. After manual correction of a certain number of discrepancies, we obtained a correlation coefficient (R^2^) equal to 0.95. Notably, these corrections were always performed on the sequence data from the AVA^®^ pipeline analysis. A previous study, based on 34 HIV-1 RT and protease sequences, also revealed a good correlation, equal to 85.0%, between AVA^®^ and SmartGene^®^ pipeline analyses [20]. However, the major issue encountered in these analyses concerned the alignment of homopolymeric regions, for which the poor quality could lead to major errors and discrepancies between the pipeline analyses. It has been well documented that this problem is specifically related to the GS Junior^®^ 454 and Ion Torrent technologies [21–24]. This technology has been discontinued by the manufacturer. Due to this limitation, when MRVs are located in homopolymeric regions, the results need to be interpreted with a degree of caution and they should be visually checked. Most of the discrepancies concerned low-level MRVs, with frequencies of 1%-2%. Indeed, when we used 2% as the threshold for MRV detection, the number of discrepancies between the various pipeline analyses was reduced by a factor of three for both the protease and the RT regions.

In the second part of this study, we compared Geneious^®^ and SmartGene^®^ pipeline analyses based on 313 MRVs obtained with an Illumina^®^ platform. This revealed excellent correlation coefficients, with R^2^ = 0.987 for protease, R^2^ = 0.995 for RT, and R^2^ = 0.993 for integrase. Of note, Illumina technology generates approximately 100 times more sequences than GS-junior technology and it does not generate a higher error rate in homopolymeric regions [22]. This higher level of coverage contributes to the high quality of the results that are obtained. Geneious^®^ software can be tailored for alignment of viral quasi-species, if the algorithm and the alignment parameters are chosen correctly in relation to the objective of the project, especially in terms of the aligner software and quality trimming. Thus, addressing the aims of this study first required becoming accustomed with using this software for this application. Although it was found to not be particularly intuitive and to require a considerable period of learning, this software nonetheless yielded reliable results. In our study, most of the differences between the Geneious^®^ and the SmartGene^®^ pipeline analyses concerned low-level MRVs, with frequencies of 1%–2%. Thus, among the 15 discrepancies observed at the detection threshold of 1%, only three remained at the detection threshold of 2%, leading to a very high level of concordance of 98.6%. Furthermore, for most of the cases of discrepancies involving MRVs with frequencies of 1%–2%, the MRVs were in fact detected by the second software program, at frequencies of 0.5%-1%, and they hence did not exhibit significant differences.

Clearly, the various analysis pipelines used different criteria for read quality trimming and alignments algorithms, since they did not retain the same number of final reads for the analyses. In addition, the HXB2 sequence was used as a reference, including for non-B subtype sequences analyses. This could have played a role in coverage, affecting the final number of reads that were analyzed and thus possibly altering the frequency of MRVs that could have resulted in discrepancies for HIV-1 of the non-B subtype.

In conclusion, due to misalignments, AVA^®^ was found to not be the most suitable software for detecting MRVs in homopolymeric regions. Geneious^®^ software, on the other hand, proved to be reliable for detecting MRVs within viral quasispecies. Although Geneious^®^ software is not expensive; it required a period of learning in order to optimize it for the analysis at hand. SmartGene^®^ software proved to be more user-friendly and it provided a time-saving, offering an integrated solution from the raw sequence data, to the results, and finally to the clinical report. All of the tested tools are web-based, thus constituting a portable work-space. The read alignments are viewable and different technical variables are readily available, thus allowing for technical validation of the results.

Our study shows that reliable detection/quantification of MRVs with frequencies of 1%-2% is not always a given, thus indicating that a degree of caution is required when operating in this range. According to our findings, the presence of MRVs with frequencies of 1%-2% should be confirmed by the analysis of reads with a second software program and after having visually checked the quality of the read alignments. In the HIV field, most of the virological studies to date that assessed MRVs opted to use 0.5% or 1% as the detection threshold, without necessarily providing details of the bioinformatics analysis and validation for low-level MRVs [5,12,25,26]. There is currently no clinical cut-off for MRV interpretation, except for the K103N NNRTI resistance mutation. The presence of more than 2,000 copies/mL of this NNRTI mutation is indicative of an increased risk of virological failure of a first-generation NNRTI-based regimen, with an odds ratio of 47.4 [27].

In this study, we found an excellent correlation between SmartGene^®^ and Geneious^®^ or AVA^®^ pipeline analyses in terms of the detection and quantification of HIV MRVs. In the context of HIV infection, for the time being, MRV assessment remains in the domain of clinical research. There is a need to accumulate data to provide clinical cut-offs of MRVs that could impact the virological response. Most importantly, our results argue for use of a 2% threshold for MRV detection, rather than the 1% threshold that is commonly used at present. Indeed, in our study, most of the discrepancies observed between the analysis pipelines concerned low-level MRV with frequencies of 1%-2%. Their technical validation is time-consuming as it requires the use of different alignments algorithms as well as visualization of the alignments.
